# Multi‐Scale Stratification of Metabolomic Profiles for Alzheimer's Subgroup Identification

**DOI:** 10.1002/alz70855_102842

**Published:** 2025-12-23

**Authors:** Mythreya Dharani, Mustafa Buyukozkan, Rima F. Kaddurah‐Daouk, Matthias Arnold, Jan Krumsiek, Richa Batra

**Affiliations:** ^1^ Institute for Computational Biomedicine, Englander Institute for Precision Medicine, Department of Physiology and Biophysics, Weill Cornell Medicine, New York, NY, USA; ^2^ Duke University, Durham, NC, USA; ^3^ Helmholtz Zentrum München ‐ German Research Center for Environmental Health, Neuherberg, 85764, Germany

## Abstract

**Background:**

Alzheimer's disease (AD) patients exhibit diverse cognitive impairments, brain atrophy patterns, and pathological hallmarks, suggesting the existence of distinct subtypes. Molecular profiling offers a powerful approach to uncovering this variability and defining clinically relevant subgroups. To fully capture the molecular landscape of each AD subtype, it is critical to explore unique profiles across omics levels. Despite advances in subgroup identification methods, most approaches treat all molecular measurements equally, limiting their ability to isolate disease‐relevant features.

**Method:**

To address this, we developed **AutoSGI**, a framework that identifies informative feature subsets to characterize patient subgroups. This is achieved using either (1) pre‐defined pathway annotations or (2) hierarchical clustering of features to generate feature subsets. Each subset is then analyzed to identify subgroups, with statistical adjustments for multiple feature subsets. For subgroup identification, we employ our in‐house toolbox, SGI (Subgroup Identification), which hierarchically clusters samples and evaluates clinical outcomes at each branch point of the hierarchical tree to identify meaningful subgroups.

**Result:**

We demonstrate the utility of AutoSGI in two case studies on AD, focusing on metabolic and lipidomic profiles to identify clinically significant subgroups. Metabolic dysregulation is of particular interest, as systemic metabolic changes have been implicated in AD brain pathology and central metabolic comorbidities. In the first case study, AutoSGI stratifies samples by disease stages using metabolomics data from postmortem brain tissue. The identified subgroups show significant differences in neuropathological outcomes, including Braak and CERAD scores. In the second case study, AutoSGI analyzes pathway enrichment scores from blood lipidomics data to uncover subgroups with varying disease progression rates. These subgroups are defined by features relevant to disease progression, including cerebrospinal fluid (CSF) tau protein levels and Alzheimer's Disease Assessment Scale‐Cognitive Subscale (ADAS‐Cog‐13) scores over 1‐2 years.

**Conclusion:**

These results highlight AutoSGI's ability to leverage multi‐scale feature subsets for robust subgroup identification, providing insights into the heterogeneity of AD and its progression.

